# Short-Chain Fatty Acids: A Soldier Fighting Against Inflammation and Protecting From Tumorigenesis in People With Diabetes

**DOI:** 10.3389/fimmu.2020.590685

**Published:** 2020-12-08

**Authors:** Qiyu Yang, Jing Ouyang, Fengjun Sun, Jiadan Yang

**Affiliations:** ^1^ Department of Radiation Oncology, Chongqing University Cancer Hospital and Chongqing Cancer Institute and Hospital, Chongqing, China; ^2^ Chongqing Public Health Medical Center, Chongqing, China; ^3^ Department of Pharmacy, Southwest Hospital, The Third Military Medical University (Army Medical University), Chongqing, China; ^4^ Department of Pharmacy, The First Affiliated Hospital of Chongqing Medical University, Chongqing, China

**Keywords:** short-chain fatty acids, gut dysbiosis, inflammation, diabetes, cancer

## Abstract

Converging evidences showed that people with diabetes mellitus (DM) have significantly higher risk for different cancers, of which the exact mechanism underlying the association has not been fully realized. Short-chain fatty acids (SCFAs), the fermentation products of the intestinal microbiota, are an essential source for energy supply in gut epithelial cells. They have been reported to improve intestinal barrier integrity, prevent microbial translocation, and further dampen inflammation. Gut dysbiosis and reduction in SCFA-producing bacteria as well as SCFAs production in the intestine are commonly seen in metabolic disorders including DM and obesity. Moreover, inflammation can contribute to tumor initiation and progression through multiple pathways, such as enhancing DNA damage, accumulating mutations in tumor suppressor genes Tp53, and activating nuclear factor-kappa B (NF-κB) signaling pathways. Based on these facts, we hypothesize that lower levels of microbial SCFAs resulted from gut dysbiosis in diabetic individuals, enhance microbial translocation, and increase the inflammatory responses, inducing tumorigenesis ulteriorly. To this end, we will discuss protective properties of microbial SCFAs and explore the pivotal roles SCFAs played in the link of DM with cancer, so as to take early precautions to reduce the risk of cancer in patients with DM.

## Introduction

Diabetes mellitus (DM) is characterized by different metabolic abnormalities, including hyperglycemia, insulin secretion deficiency, insulin dysfunction, and energy metabolism disturbances (1, 2). The World Health Organization (WHO) reported about 422 million people have DM in the world. Morbidity and prevalence of DM have been steadily rising over the past few decades. Aside from DM, cancer ranks as the second main cause of death in the developed world and the third cause for death in developing nations (3). DM and cancer share several common characteristics, such as microbial imbalance and bacterial translocation (4–6). Unfortunately, these two diseases are frequently coexistent and can affect each other, worsening the prognosis of patients.

Generally compared with non-diabetic people, the incidence of cancer in diabetic patients increased by approximately 20–25%, depending on specific cancer type and site (7, 8), which has been observed in both Asian and Western populations (9). People with diabetes accompanied by cancer also experienced higher mortality risk, in contrast with the group with only cancer (10). Practically speaking, when comparing to the general population, the significantly increased risk of cancer in diabetic individuals has been proved in hepatocellular and pancreatic carcinomas, which were 2.5 and 1.94 times higher respectively (11, 12). Friberg et al. demonstrated that in diabetic women, the risk for endometrial cancer nearly doubled (13). The epidemiological literature indicates that patients with DM are at modestly increased risk (by 19–42%) of developing colorectal, gastric, kidney, breast, and bladder cancers (14–16). Numerous studies have also declared the positive associations of DM with non-Hodgkin lymphoma, leukemia, and myeloma, which is about 19–22% higher in patients with only type II DM (17–19). Regarding type I DM cohorts, in comparison with the non-diabetics, increased risk of several cancers might be reported in multiple research cohorts, yet inconsistent if all studies are considered (20–22).

However, regarding the existing correlation of DM with cancer, the mechanisms remain to be established. Identifying and utilizing the intermediate links between the two diseases are essential to reduce the risk of cancer and improve the symptoms of DM. Short-chain fatty acids (SCFAs), as functional microbial metabolites (23), might be a possibility to unlock the tie. Herein, we will go further behind this correlation and focus on SCFAs, which may be involved in the progress of DM developing into cancer.

## Hypothesis

**Figure 1:**
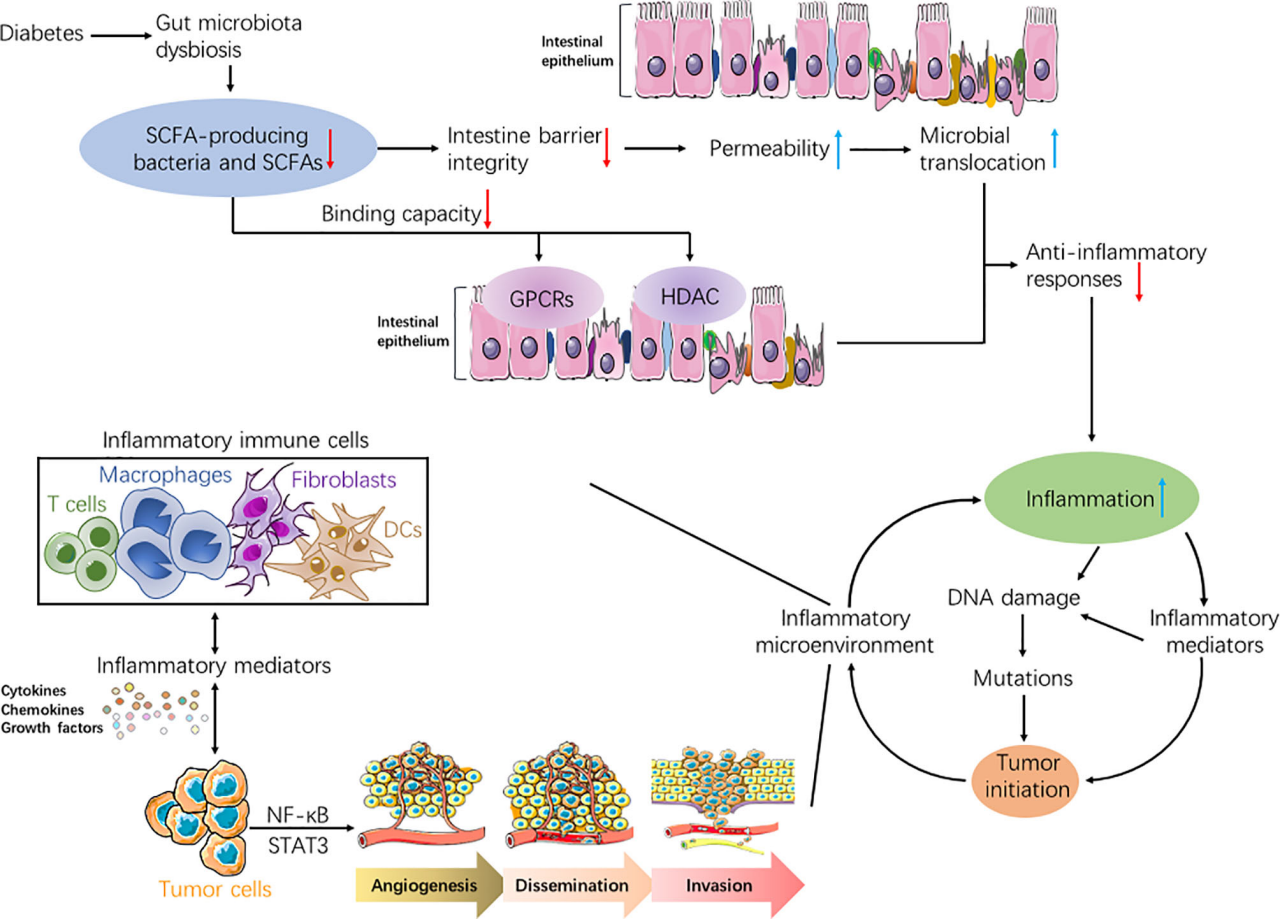
Flowcharts representing the association of diabetes, short-chain fatty acids (SCFAs), inflammation, and tumor. Due to gut microbiota dysbiosis in people with diabetes, both SCFAs-producing bacteria and the produced SCFAs decreased. Reduction in SCFAs could weaken anti-inflammatory responses in diabetics through diverse mechanisms, such as increasing gut permeability, inducing microbial translocation, and attenuating binding capabilities of SCFAs to both GPCRs and HDAC. Hence, the inflammation was exacerbated in diabetic patients. Increased inflammation may lead to tumorigenesis in normal cells through accumulated genetic mutations, which proceeded from DNA damage. Moreover, various inflammatory immune cells can secrete abundant inflammatory mediators such as cytokines, chemokines, and growth factors, which may activate key transcription factors, including NF-κB and STAT3. This could initiate tumor growth in normal cells, or boost malignant processes in tumor cells including angiogenesis, dissemination, and invasion. In turn, inflammatory reactions were aggravated under sustained tumor-associated inflammation, thus forming a vicious and positive feedback loop between inflammation and tumor.

Based on recent evidences, we hypothesize that lower abundance of SCFAs resulted from gut microbial dysbiosis in diabetics could induce the destruction of the gut mucosal barrier, increase microbial translocation, and promote inflammation responses, further boosting inflammatory-malignant transformation and enhancing risk of tumorigenesis (**Figure 1**).

## Gut Microbiota Dysbiosis and Lower SCFAs in DM

The intestinal barrier is the functional defense line, consisting of microbiota, intestinal epithelial cells (IECs), and mucosal immunity (24). The gut microbiota are collectively referred to as eukaryotes, archaea, and bacteria, among which bacterial phyla are the most abundant (25). Investigations identified that keeping the diversity and quantity of human gut microbiota, which can protect the host against pathogens by competition for niches and nutritive supply, improvement of immune functions, and regulation of metabolism, is of critical importance in maintaining intestinal homeostasis (26, 27). *Akkermansia muciniphila* was reported to improve intestinal epithelial monolayer integrity by stimulating colonic mucin secretion, increasing the thickness of the gut mucus layer, and binding directly to intestinal cells (28, 29). On the contrary, dysregulation of gut microbiota composition, also called dysbiosis, could impair the balance between the commensal species and various pathogens, as well as decrease the release of metabolic SCFAs and antimicrobial molecules such as bacteriocins (30). Depleted Firmicutes and increased Proteobacteria such as *Enterobacter cloacae* and *Enterobacter* species contribute to the disruption of IECs, which in turn induces the translocation of intestinal microbiota and their toxins, leading to infectious threats (31, 32). Dysbiosis may also regulate IECs to release intestinal miRNAs, which could orchestrate the immune responses *via* TLR dependent pathways or PRR families to fight against pathogens (33). Other than SCFAs and bacterial toxins, microbiota might influence the intestinal barrier through other signal pathways, including bile acids metabolism and endocannabinoid system (34, 35).

Emerging evidences indicated DM is significantly correlated with gut microbiota dysbiosis, which harms the integrity of the gut wall and promotes the shift of endotoxemia from the intestinal cavity into the circulatory system, hence triggering inflammation, autoimmune responses, and oxidative stress (36). Larsen et al. demonstrated that microbial dysbiosis could provoke changes in composition and distribution of gut microorganisms, particularly intestinal bacterial species in the mucosa, and metabolic activities (37). Compared to non-diabetics, *Lactobacillus spp* and Betaproteobacteria groups were highly enriched in diabetics, which was positively connected to plasma glucose and could trigger inflammation effect in DM (38–40). Considerable studies indicated that the abundance of *Akkermansia muciniphila*, Firmicutes, and *Clostridium* spp was significantly decreased among individuals with DM, inducing unfavorable effects on nutrient metabolic control, glucose tolerance, and inflammation responses (41–43). In addition, the diabetics possessed lower abundance of SCFA-producing organisms, including *Roseburia intestinalis* and *Faecalibacterium prausnitzii*, which led to reduced anti-inflammatory SCFAs levels, especially butyrate (37, 44, 45). Similar results have been observed in animal studies describing that the levels of SCFA-producing bacteria and SCFA production declined remarkably in diabetic mice versus the diabetes-free group (46, 47). Deficiency in SCFA production could promote intestine microbes to spread into the systemic circulation, thereby inducing or aggravating systemic inflammatory responses (48, 49).

Notably, intestinal SCFA producers and their metabolites SCFAs are reported to play crucial roles in the pathophysiology of DM. Compared to healthy controls, type 1 diabetes mellitus (T1DM) patients harbored decreased population of SCFA-producing bacteria and circulating SCFAs, in line with markedly decreased genes contributing to SCFAs synthesis, which is associated with disturbed microbiota composition (50–52). Gut metagenome data also revealed that subjects with type 2 diabetes mellitus (T2DM) were characterized by a decrease in SCFA-producing microbiota and SCFA abundance, issued from altered gut microbiota, impaired intestinal barrier, and increased plasma levels of lipopolysaccharides (LPS) (53–55). For diabetics, the favorable effects of SCFAs were mainly identified in reducing serum glucose levels, improving insulin resistance, mitigating inflammation, and enhancing secretion of protective glucagon-like peptide 1 (GLP-1) (56–58). Conversely, dietary interventions with SCFAs were shown to alleviate T1DM in mice models, as evidenced by decreased auto-immune T cell counts, suppressed B cell proliferation, and expanded autoimmune FoxP3+ Treg cell repertoire in the colon, spleen, and systemic lymph nodes (46, 58). By providing SCFA-releasing diets to T2DM patients, it could significantly improve metabolic disorders by enriching SCFA-producing microbes mass, ameliorating individual glucose tolerance, and reducing levels of hemoglobin A1c (59–61). Herein, we discuss the latest developments in the protective effects of SCFAs and appreciate their potential involvement in diseases.

## Effects of SCFAs On Inflammation, Immunity, and Metabolism

SCFAs, predominantly acetate, butyrate, and propionate, are anaerobic fermentation metabolites of fiber produced by intestinal microorganisms (62). SCFAs act as the principal energy source for colorectal cells, enhancing intestine epithelium integrity (63). Besides, as leading messenger molecules between gut microbiota and host health, SCFAs can enter the blood circulation, protect the gut barrier, regulate inflammatory responses, and influence immune functions of distal tissues (64).

SCFAs can promote the intestinal epithelium function, mainly by hypoxia-inducible factor-1 (HIF-1), which is a transcription factor stabilizing intestinal epithelial barrier (65). Antibiotic-mediated microbiota depletion reduces intestinal levels of HIF and causes epithelium impairment, which could be restored by supplementing butyrate. However, the effects of butyrate on gut epithelial barrier protection vanished when lacking HIF (29). In addition, SCFAs can restore the intestinal barrier integrity by inducing gene expressions of inter-epithelial junction protein Claudin-1, and activating other transcription factors such as STAT3 and SP1, even in inflammatory conditions (66, 67). Another involved mechanism is that SCFAs could induce IECs to produce antimicrobial peptides (AMPs), recruit neutrophils and anti-inflammatory cytokines, suppress activation of NLRP3 inflammasome, and further protect the intestinal barrier from damage by pathogens (68, 69).

When SCFAs were supplemented *in vivo*, they were shown to preserve the molecular barrier of intestinal epithelium. Vieira et al. reported that oral SCFAs could significantly improve intestinal trophism and inhibit leukocytes and other immunocytes infiltration, further attenuating the inflammatory state of gut barrier in rats with acute ulcerative colitis (70). SCFAs treatment may also constitute substrates for gut mucosa and ameliorate intestinal damage, by restoration of glutathione levels and reduction in proinflammatory mediators, including nitric oxide (NO) and tumor necrosis factor-α (TNF-α) in rat models of colitis (71). In addition, rectal administration of SCFAs could promote mucus secretion and intestinal repair, by increasing abundance of mucus-associated bacteria and expression of genes required for pathogens elimination, such as IL-17A and IL-1β in mice with enteritis (72, 73).

Säemann et al. reported that administration of SCFAs could not only reduce secretion of pro-inflammatory markers such as TNF-α, IL-6, and IL-12 but also increase release of anti-inflammatory markers including IL-10 in cultured human peripheral blood mononuclear cell (PBMCs) *in vitro* (74), providing important clues to cognize the anti-inflammatory actions of SCFAs. With evidence-based potentiated generation of anti-inflammatory regulatory T cells, inhibited release of reactive oxygen species, and suppressed production of pro-inflammatory cytokines including TNF-α and IL-1β, anti-inflammatory effects of dietary SCFAs have been confirmed in different animal models, such as steatosis, allergic airway inflammation, and chronic kidney disease (CKD) (64, 75, 76). In addition, clinical investigations revealed that the inflammatory potential, analyzed by the TNF-α/IL-10 ratio, was significantly higher in the diabetic group than the healthy ones, which may be counteracted by dietary fiber that can produce SCFAs (77, 78), corroborating the inflammation inhibitory potential of SCFAs. In most cases, reduced inflammatory conditions and improved immunity are inseparable.

Several mechanisms have already been explored on the anti-inflammation influences of SCFAs (1). Permeability: Increased permeability is believed to induce microbial translocation, which could trigger an inflammatory cascade (79). Chen et al. confirmed that the representative SCFA, butyrate, is a crucial substrate for promoting epithelial cell growth, which can maintain the colonic epithelium, prevent excessive gut permeability, and even induce innate immune responses to injury and invasive microorganisms if necessary (80) (2). Nuclear factor-kappa B (NF-κB): NF-κB is a critical transcription factor for inducing expressions of multiple inflammation related genes. Studies have shown that butyrate can inhibit NF-κB activation in human macrophages and epithelial cells (81, 82) (3). Histone deacetylation (HDAC): HDAC inhibitors were initially developed as cancer-combating agents. Nowadays HDACs inhibitors are attracting much more interest as anti-inflammatory agents, independent of their known proapoptotic or cell cycle arrest actions on malignant cells (83). SCFAs are natural HDACs inhibitors, facilitating expressions of anti-inflammatory genes in the immune cell, promoting T lymphocytes as they differentiate into effector T cells such as Th1 and Th17 cells subsets, and boosting their immune responses in inflammation (84, 85). Kim et al. also revealed that by promoting activation and differentiation of B cells to plasma B cells, SCFAs accelerated the production of most antibodies types, including IgG and IgA (86) (4). G-protein coupled receptors (GPCRs): SCFAs could activate GPR41 and GPR43 in intestinal epithelial cells, leading to transmission of mitogen-activated protein kinase signaling, and rapid secretion of chemokines and cytokines (87, 88). Singh et al. showed that *via* activating GPR109A in macrophages and dendritic cells, SCFAs make them highly efficient inducers of regulatory T cells, particularly FoxP3^+^ T cells, to limit inflammation and control carcinogenesis (88). So SCFAs not only act as anti-inflammatory bacterial metabolites but also function as immune boosters to prepare the host to better exterminate pathogens.

Furthermore, SCFAs are among the most extensively studied microbial metabolites that intervene in host metabolism. By binding to GPR43 and GPR41, SCFAs are able to raise plasma levels of GLP-1, peptide YY (PYY), and leptin, resulting in reduced food intake, enhanced glucose metabolism, and improved glucose homeostasis (89–91). Fascinating animals researches also indicated that butyrate and propionate may both activate gene expression germane to intestinal gluconeogenesis, through cAMP-dependent pathway and GPR41-dependent gut-brain circuits respectively, eventually improving glycaemia control and ameliorating insulin sensitivity (92, 93). Another vital function of SCFAs is intracellular metabolic integration to produce energy such as adenosine triphosphate (ATP) (94). Canfora et al. demonstrated that infusions of SCFA mixtures in the colon boosted energy consumption, enhanced fat oxidation, and decreased lipolysis in metabolic profiles (95).

In order to achieve these benefits, the production of SCFAs can be encouraged in various ways, such as natural sugar and high-fiber intake (96, 97). However, it is necessary to determine the dosage range of SCFAs before they can be applied in clinic, as superfluous SCFAs might exert some adverse effects, such as accelerating cholesterol synthesis and lipid accumulation within the liver (98, 99). Excessive acetate and butyrate may also be involved in host fat storage by enhancing energy intake and intestinal polysaccharide degradation, thereby contributing to weight gain and obesity phenotype in obese mice (100, 101).

Considering their extreme importance in resisting inflammation in the gut, SCFAs are regarded with mighty potential for certain diseases including metabolic conditions and cancer, providing strong theoretic foundation to launch SCFAs-based clinical trials for cancer treatment. However, the causative role of SCFAs abundance in diabetics with respect to carcinogenesis needs further elucidation.

## Roles of Inflammation and SCFAs in The Context of Cancer

Inflammation usually involves the activation, recruitment, and functioning of innate and adaptive immune cells, which is essential for the host to defend against pathogens, repair damaged tissues, and regulate tissue homeostasis (102). Acute inflammation is protective and normally self-limited, which would be terminated after elimination of harmful triggers or completion of the restorative process (103). This self-limiting property of acute inflammation was also verified by Bannenberg and his colleagues in mouse peritonitis models, induced by zymosan (104). Once acute inflammatory responses are out of control and cause tissue damage, it will amplify inflammation and progress to chronicity (105), hence predisposing people to cancer development. More importantly, inflammatory immune cells together with fibroblasts and vascular endothelial cells constitute the stroma network for cancer cell survival, namely the tumor microenvironment (TME) (106), which can boost tumor occurrence, tumor promotion, malignant transformation, and metastatic transmission (107, 108). In general, tumor extrinsic inflammation can be caused by certain factors including infection, metabolic diseases, autoimmune diseases, and smoking, while tumor intrinsic inflammation can be triggered by cancer-related gene mutations or by recruiting and activating inflammation-fighting cells (109). Inflammation, irrespectively of its inducement or appearance, owns a significant effect on carcinogenesis. Typical pro-inflammatory cytokines IL-17 and IL-23, which can promote tumor development and progression respectively, were both upregulated in mouse models of colorectal cancer (CRC) (110, 111). In turn, tumorigenesis may enhance pro-tumorigenic inflammation, illustrating the essential circle between inflammation and cancer. Collectively, epidemiological studies showed that inflammation is linked to the initiation of around 20% of cancers (112). To be specific, chronic inflammations caused by the hepatitis virus and *Helicobacter pylori* are associated with the majority of hepatocellular and gastric carcinomas respectively (113–115). Approximately 2% of CRC develops in patients with ulcerative colitis (UC) (116, 117). Chronic airway inflammations with tobacco smoke and airborne particulates are the major risk factors for lung cancer (118, 119).

Although the predisposing factors or sources may vary, inflammation always goes together with increased risk of cancer, oncogenic transformation, and malignant progression *via* multiple approaches (1). Enhancement of DNA damage. During inflammatory conditions, both inflammatory and epithelial cells can release chemicals like reactive oxygen and nitrogen species (RONS), which may cause DNA lesions (120). Furthermore, the generated DNA damage signals and cytotoxicity can promote inflammation, inducing a continuous vicious circle between DNA lesions and DNA repair, which can further enhance DNA damage. Subsequently, this feedback loop induces genetic mutations, genome instability, and eventual tumorigenesis (121, 122). Chen et al. reported that 7,12-Dimethylbenz[a]anthracene (DMBA), a mighty genotoxic agent, could activate the cGAS–cGAMP–STING pathway, inducing inflammation-driven cutaneous carcinogenesis in mouse models (123). Singhal et al. have manifested that DMBA was engaged in mammary carcinogenesis as well as the distant metastasis in skin-tumor-sensitive (STS) female mouse strains, mimicking DMBA‐induced human breast cancer (124) (2). Inactivation of anti-oncogene and activation of oncogenes. Without additional inducers, chronic inflammation can induce accumulated mutational in-activations of tumor suppressor genes such as Tp53 in the epithelial cells, leading to upregulation of microtubule modulin stathmin1 and increased chemoresistance in breast and colon cancer cell lines or patient specimens (125–127). Moreover, accumulation of activated mutations in epithelial oncogenes (K-ras and c-Myc) can be promoted by inflammation-induced DNA damage or inflammatory cytokines, which can also cooperate with other inflammatory stimuli to manifest cancerogenic activities, such as enhancing mutagenesis, promoting tissue injury, and ultimately carcinogenesis (128, 129) (3). Regulation of signaling pathways. Both NF-κB and signal transducer and activator of transcription 3 (STAT3) have been identified as cardinal inflammatory signaling molecules during carcinogenesis. Survival signals activated by NF-κB and STAT3 were enhanced in mutated epithelial cells, which protected cells against the attack of cytotoxic T lymphocytes, boosted malignant clones, and enabled tumor outgrowth (130–132). Otherwise, programmed cell death protein-1 (PD-1) pathway was activated to deliver inhibitory signals, which may be evoked by inflammatory signals interferon‐gamma (IFN‐γ), K-ras mutations, or K-ras/Tp53 co-mutations. This could drive T cell exhaustion, generate a tolerant microenvironment, and help cancerous cells escape immune surveillance and survive, particularly for patients with lung adenocarcinoma (133–135) (4). Modulation of inflammatory mediators. Cytokines, chemokines, and growth factors are the predominant cell-signaling molecules produced by multiple cells in the inflammatory microenvironment and are fundamental to tumor development in different stages (136). Dash reported that TNF-α was among the chief cytokines in inflammatory responses, which rendered epithelial to mesenchymal transition (EMT), accelerated cancerous cells invading process, and elicited other inflammatory proteases to orchestrate inflammatory conditions (137). Tumorigenic cytokines such as IL-6 and IL-11 could directly act on tumor cells, enhancing cell proliferation, stimulating angiogenesis, and expanding cancer stem cell (CSC) population, in mouse xenograft models of gastrointestinal and breast cancers (138, 139). Calon et al. identified that IL-11 was also involved in summoning myeloid cells such as fibroblasts and tumor growth factor-β (TGF-β), which could facilitate cancer cell migration, promote tumor invasion, and assist metastatic transition in colorectal carcinoma of mice (140). Regarding the similarity of inflammatory processes in distinct cancers, preventing or controlling inflammation may be an imperative treatment for cancer.

Numerous studies have demonstrated that some bacteria can secrete tumor-boosting metabolites like secondary bile acids, whereas some other species can generate tumor-suppressing metabolites such as SCFAs (141, 142). Echoing previously demonstrated anti-inflammation significance of SCFAs, substantial epidemiological data established that increased incidence of inflammatory diseases and cancer was linked to subjects with diets poor in SCFAs or decreased concentration of fecal SCFAs, typically for gastric and breast cancers (143–145). SCFAs in the gut and other organs can extensively reduce carcinogenesis as well as prevent and treat gastrointestinal and lung cancers, by inhibiting cell growth and migration, suppressing histone deacetylase, and inducing apoptosis (146–148). Specifically, Ohara et al. reported that increased CRC risk correlated tightly with altered gut microbiota, reduced output of SCFAs, and worse inflammation state (149). Therefore, increasing SCFAs production *via* regulation of gut microbiota should have a bright future in anti-cancer therapy.

Other than SCFAs, evidences also indicate that low-dose aspirin could reduce the risk of tumorigenesis, particularly in CRC. As an inhibitor of cyclooxygenase (COX)-1, aspirin exerts its significant chemo-preventive effects in CRC through inhibition of NF-κB dependent pathways, Wnt/β-catenin signaling, and additional COX proteins acetylation (150, 151). The notable anti-tumor effects of aspirin have been observed by reduction in platelet activation, inhibition of tumor angiogenesis, and decrease of pro-inflammatory agents (152). In addition, low-dose aspirin could reduce metastasis in colon cancer, mainly due to its effective inhibition of prostaglandin E2 (PGE2) formation and platelet-tumor cell aggregation (153). Different from aspirin and other modulators of NF-κB, SCFAs could not only inhibit NF-κB signaling pathway but also promote gut microbial ecology and enhance intestinal integrity, further fighting against inflammation and reducing tumorigenesis through multiple pathways. Moreover, as SCFAs can be supplemented by dietary interventions, they were considered to be safer and more obtainable. However, aspirin can cause several severe adverse effects, such as gastrointestinal hemorrhage, intracranial bleeding, and hypersensitivity reactions (154, 155). Even aspirin in a low dose can lead to bleeding in diabetics (156). These advantages push SCFAs to be potential agents to lower cancer risk.

## Conclusion

Low levels of SCFAs, fermented by gut microbiota, have been linked with DM-related intestinal barrier dysfunction, gut dysbiosis, and aggravated inflammation. Mostly diabetic patients exhibited higher incidence of various tumors. In addition, an inverse relationship between decreased production of SCFAs and increased risk of cancer has also been discovered, showing that SCFAs have the potential of associating DM with tumorigenesis. Accordingly, diets high in fiber and transferring fecal microbiota, aiming at increasing SCFAs production or strains of SCFA-producing species, can attenuate the progression of inflammatory disorders by altering gut microbiota composition and suppressing inflammation (157), which may provide a new paradigm in cancer prevention and treatment in diabetes. However, the effectiveness and security of utilizing SCFAs to dampen inflammation and decrease the incidence and mortality of tumors in diabetic individuals require more research to confirm. Immediate evidences are still needed to validate and recognize the favorable influences of SCFAs as a capable regulator of cancer-related inflammation in DM. Efforts of collaboration encompassing metabolism, microbiology, immunology, oncology, and dietotherapy will define routes of administration and dosage to obtain the optimum benefits of SCFAs as an anti-inflammatory and anticarcinogenic soldier in DM.

## Author Contributions

QY and JO wrote the first draft of the manuscript. FS provided critical revision of the manuscript. JY conceived and designed the manuscript. All authors contributed to the article and approved the submitted version

## Funding

Our work was funded by Chongqing Basic and Frontier Research project (grant number: cstc2018jcyjAX0652) and Chongqing Technological Innovation and Application Development project (grant number: cstc2019jscx-msxmX0190).

## Conflict of Interest

The authors declare that the research was conducted in the absence of any commercial or financial relationships that could be construed as a potential conflict of interest.
